# Action mechanism of early cerebral injuries after spontaneous subarachnoid hemorrhage by silence Ghrelin and angiogenic factor with G-patch and FHA domain 1

**DOI:** 10.1080/21655979.2022.2037373

**Published:** 2022-03-08

**Authors:** Jianxun Tang, Ligang Hu, Feng Long, Jie Zhang, Jingfeng Tang

**Affiliations:** a Department of Cerebrovascular Disease, The Second Affiliated Hospital of Guilin Medical University, Guilin, China; b Department of Cerebrovascular Disease, General Medical College of Guilin Medical College, Guilin, China

**Keywords:** Ghrelin, angiogenic factor with G-patch and FHA domain 1, subarachnoid hemorrhage, early cerebral injuries, oxidative stress

## Abstract

The objective of the research was to investigate action mechanism of oxidative stress and cerebral injuries after subarachnoid hemorrhage (SAH) by Ghrelin and angiogenic factor G-patch and FHA domain 1 (Aggf1) and offer new research ideas to SAH clinical treatment and SAH-induced early cerebral injuries. SAH rat models were prepared by prechiasmatic anterior cistern injection. Specific Ghrelin and Aggf1 small interfering ribonucleic acid (siRNA) were designed and injected into silence Ghrelin or Aggf1 in rat left lateral ventricles. Rats were divided randomly into sham-operated (sham), SAH model, negative control siRNA, Ghrelin silence (Ghrelin^(-/-)^), and Aggf1 silence groups. Changes of rat neurological impairment, encephaledema, cerebral tissue phosphorylated protein kinase (p-Akt), and content changes of caspase-3 protein and oxidative stress indexes were observed, including glutathione (GSH) and oxidized glutathione (GSSG). Results showed scores of neurological impairment and water content in SAH model group were reduced compared with sham group, while p-Akt protein and GSH contents were enhanced. However, caspase-3 protein and GSSG contents were declined, showing statistically meaningful difference (*P* < 0.05). Compared with SAH model group, scores of neurological impairment, cerebral tissue water content, and caspase-3 protein and GSSG contents in silence Ghrelin and Aggf1 groups were increased, while p-Akt protein and GSH contents were decreased, demonstrating statistically meaningful difference (*P* < 0.05). To conclude, silence Ghrelin and Aggf1 aggravated early cerebral injuries after SAH, revealing that Ghrelin and Aggf1 could protect brains to some degree.

## GRAPHICAL ABSTRACT

**Figure:**
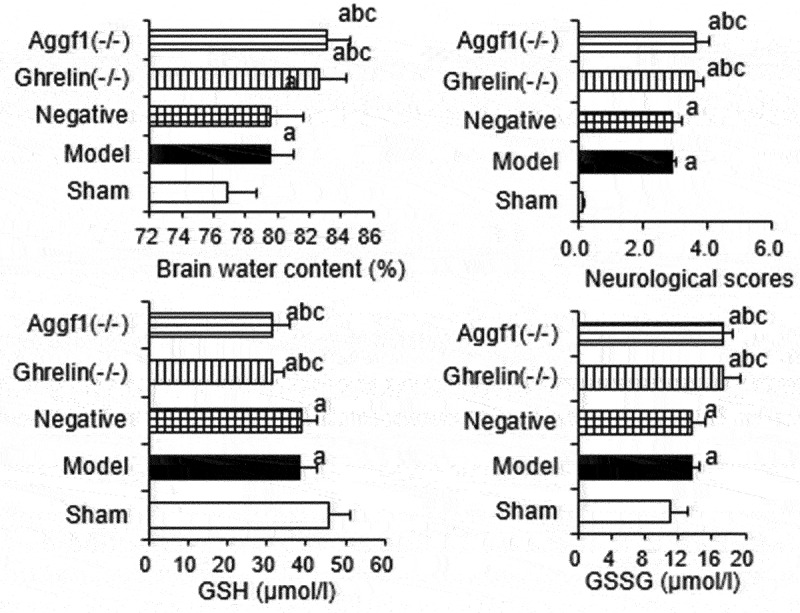

## 1. Introduction

Subarachnoid hemorrhage (SAH) is a cerebrovascular disease posing a serious threat to human life and health. About 10% of stroke patients suffer from SAH with mortality and disability both reaching as high as 50% [1,2]. Cerebral injury is the main factor resulting in the death or poor prognosis of SAH patients after treatment. Neuroinflammation reaction is the major action mechanism causing cerebral injury after SAH [3,4]. Angiogenic factors with G-patch and FHA domain 1 (Aggf1) can promote the proliferation of endothelial cells and the generation of capillaries [5]. Relevant studies revealed that angiogenic factors of Aggf1 got involved in vascular development and angiogenesis and played a critical role in AngII-induced angiogenesis [6]. Wang et al. (2021) [7] discovered that angiogenic factors, including AGGF1 and VEGFA (vascular endothelial growth factor (VEGF) A) both could promote angiogenesis, regulate the proliferation and migration of endothelial cells, and promote hematopoiesis and vascular development [7]. In addition, Aggf1 proved to inhibit inflammation reactions. Ghrelin was the first endogenous ligand that secreted hormones of growth hormones discovered by human beings, and it primarily originated in gastrointestinal tract [8]. Based on more in-depth studies, Ghrelin in animal models could protect cerebral nerve by action mechanism, including antioxidant, anti-inflammatory reaction, and antiapoptosis [9,10]. Some researchers established mice cerebral hemorrhage model and investigated the action mechanism and potential molecular mechanism of Ghrelin in secondary brain injury (SBI) after intracerebral hemorrhage (ICH). Besides, they found out that Ghrelin activated and promoted Nrf2/ARE signal pathways to avoid ICH-induced SBI by inhibiting NLRP3 inflammasomes [11]. Ghrelin showed the potential in promoting angiogenesis to improve endothelial function by Jagged1/Notch2/VEGF. By now, whether Aggf1 could alleviate neuroinflammation reaction after SAH still needs to be investigated and verified, and the protection of early cerebral injured nerve after SAH by Ghrelin should also be investigated by a large number of studies [12,13].

Experimental rats were divided into sham-operated (sham) group, SAH model group, negative control group, Ghrelin silence (Ghrelin^(-/-)^) group, and Aggf1 silence groups to explore mRNA expression levels of Ghrelin and Aggf1 in cerebral tissues of rats in different groups and analyze the influences of Ghrelin and Aggf1 silence on rat neurologic dysfunction scores and rat brain water contents. Besides, Western blotting was adopted to detect the expression levels of Ghrelin, Aggf1, p-Akt, and caspase-3 in rat cerebral tissues. GSH contents, GSSG contents, and the differences in ratio of GSH/GSSG in rat cerebral tissues were compared to analyze the influences of silence Ghrelin and Aggf1 on rat cerebral oxidative stress, which confirmed that Ghrelin and Aggf1 could resist oxidation to some degree and avoid early cerebral injury after SAH. The objective of the research was to provide the theoretical reference for the selection of therapeutic targets of subsequent cerebral injuries after SAH.

## 2. Materials and methods

### 2.1. Experimental materials

102 healthy adult male Sprague-Dawley (SD) rats were purchased from Kaixue Biotechnology (Shanghai) Co., Ltd. Rat anti-Ghrelin monoclonal antibody (31901ES60, 100 µL), rabbit anti-rat Aggf1 polyclonal antibody (31002ES40, 100 µL), rat anti-protein kinase B (Akt)/phosphorylated protein kinase (p-Akt) monoclonal antibody (31002ES60, 100 µL), rat anti-rat caspase-3 polyclonal antibody (31001ES20, 100 µL), and rat anti-β actin monoclonal antibody (310030ES60, 100 µL) were purchased from Yeasen Biotechnology (Shanghai) Co., Ltd. Besides, protein quantification kits and efficient chemiluminescence (ECL) kit were purchased from Beyotime biotechnology Co., Ltd. Primescript™ real time (RT) reagent kit with gDNA eraser (perfect real time) and TB Green® premix ex taq™ (tli RNaseH plus) were purchased from Takara company. In addition, glutathione content detection kits were purchased from Nanjing Jiancheng science and technology Co., Ltd.

### 2.2. Experimental grouping

A total of 102 rats were included in the experiment. Before the experiment, rats were fed in animal laboratory. The average temperature was 22 ± 3°C, and average relative humidity was 55 ± 5%. In addition, they were exposed to lighting for 12 hours and allowed to eat freely. Optic chiasma blood pool injection method was utilized to prepare SAH model. After the modeling, two rats were killed to determine the successful modeling. After the successful modeling, 100 remaining rats were randomly divided into 5 groups as follows. The first one was sham group containing 20 rats. The rats in this group was injected with 0.25 mL 0.9% of stroke physiological saline solution into front cistern optic chiasma. The second one was SAH model group containing 20 rats. 0.25 mL of autoblood without heparinization was extracted from rats’ phrenicartery and then injected into optic chiasma front cistern. The third one was negative control group containing 20 rats. The left lateral ventricles of rats in the group were injected with contrast small interfering ribonucleic acid (siRNA) 24 hours before the modeling. The fourth one was Ghrelin group (Ghrelin^(-/-)^ group) containing 20 rats. The left lateral ventricles of rats in the group were injected with Ghrelin siRNA segments 24 hours before the modeling. The fifth one was Aggf1 silence group (Aggf1^(-/-)^ group) containing 20 rats. The left lateral ventricles of rats in the group were injected with Aggf1 siRNA segments 24 hours before the modeling. The implementation of the research had been approved by Hospital Animal Ethics Committee.

### 2.3. Preparation of animal models

Optic chiasma blood pool injection method was adopted to prepare SAH models [11]. Rats were weighed before surgery and then injected with 350 mg/kg 3.5% of chloral hydrate solution for anesthetization. After that, rats were fixed in prone position on cerebral stereotaxic locators. After rats’ brains were sterilized, they were cut lengthwise along the midline of heads to expose skulls, and a hole was dug 7.5 mm in front of the central line of front halogens and meninges were picked. Next, the needle was moved forward along sagittal plane, and bone wax was utilized to seal the puncture hole once the needle was inserted into sagittal plane for 10 mm at 45° to the coronal plane. Then, 0.25 mL of femoral arterial blood without heparinization was injected. After 10 seconds, bone holes were sealed by bone wax. Next, rats’ scalps were sterilized and sutured. During the whole surgery on rats, their body temperature was kept at about 37°C, and they were placed into clean rat cages after waking up from anesthesia.

### 2.4. Design and injection of siRNA

A total of 3 different Ghrelin as well as Aggf1 siRNA fragments and 1 control siRNA were designed and synthesized, respectively, which are demonstrated in Table 1. The stereotaxic locator was utilized to determine the drilling point 24 hours before modeling. Besides, 5 μL of Ghrelin and Aggf1 siRNA were taken by micro-injectors to be injected at a constant rate of 0.5 μL/min. The needle was kept at the drilling point for 5 minutes and then the needle hole was sealed by bone wax. After that, skin was sterilized and then sutured. After a 24-hour observation, SAH models were prepared.

**Table 1.** siRNA primer information

Gene name	Direction	Sequence (5’→3’)
Ghrelin-1	Sense sequence	CCUGCUGACUUACAAAUAAAU
	Anti-sense sequence	UUAUUUGUAAGUCAGCAGGAG
Ghrelin-2	Sense sequence	GGUUCAAUGCUCCCUUCGAUG
	Anti-sense sequence	UCGAAGGGAGCAUUGAACCUG
Ghrelin-3	Sense sequence	GCUUCUGCCUCCUCUGCAACU
	Anti-sense sequence	UUGCAGAGGAGGCAGAAGCUG
Aggf1-1	Sense sequence	GGUCAAGUUCAGACUAUUACU
	Anti-sense sequence	UAAUAGUCUGAACUUGACCAA
Aggf1-2	Sense sequence	GGAAGAUGUUGGAGAAGAUGG
	Anti-sense sequence	AUCUUCUCCAACAUCUUCCGG
Aggf1-3	Sense sequence	GCAGAGUCCGUAUGUUCAAGC
	Anti-sense sequence	UUGAACAUACGGACUCUGCUG
Control	Sense sequence	UUCUCCGAACGUGUCACGUTT
	Anti-sense sequence	ACGUGACACGUUCGGAGAATT

### 2.5. Modeling evaluation indexes

The neurologic function of rats in each group was evaluated in three dimensions, including diet, activity, and dysfunction 24 hours after modeling. The total score was 6 points. A higher score indicated severe neurologic function injuries of rats. Table 2 shows the evaluation standard.

**Table 2.** Evaluation standard for neurologic dysfunction

Dimensions	Specific evaluation
Diet	Ate all food (0 point); Ate some food (1 point); Not eating (2 points)
Activity	Complete free activity (0 point); Activity after being stimulated (1 point); Rare activity (2 points)
Dysfunction	No dysfunction (0 point); Unstable walking (1 point); Unable to walk (2 points)

The neurologic function of rats in each group was scored 24 hours after modeling. After being weighed, rats were anesthetized by being injected 350 mg/kg 3.5% of chloral hydrate solution. Next, rats were placed at prone position and their chests were opened to expose hearts. After that, the infusion needle was inserted before right atrial appendages were cut and processed by 0.9% of infused saline solution. Then, their brain tissues were taken out after decapitation, and other tissues on the surface of brain tissues, brainstems, and cerebellums were removed. After that, their brain tissues were weighed and then placed into ovens to be baked at 60°C for 48 hours. After baking, they were weighed again. Finally, cerebral water content was calculated according to the equation, which was (wet weight of brain tissues-dry weight of brain tissue)/wet weight of brain tissues ×100%.

### 2.6. Real-time fluorescence quantification polymerase chain reaction (PCR) detection

100 mg of brain tissues were extracted and added with 1 mL of RNAi Plus solution for homogenization. Next, it was placed at room temperature for 5 minutes before being centrifuged at 4°C and 12000 rpm for 5 minutes. After that, the extracted supernatant was added with 200 μL of chloroformic solution and then oscillated until it became even. Then, it was placed at room temperature silently for 5 minutes before being centrifuged at 4°C and 12000 rpm for 20 minutes. The extracted supernatant was added with isopropanol of equal volume and then mixed evenly. Next, it was placed at room temperature silently for 10 minutes. After that, it was centrifuged at 4°C and 12000 rpm for 10 minutes. Then, supernatant was removed and 1 mL 75% of the precooled ethyl alcohol solution was added. After being washed and deposited, it was centrifuged at 4°C and 12000 rpm for 5 minutes. Then, supernatant was removed, and it was dried before a proper amount of RNA-free ultra-pure water was added to dissolve sediments. After the concentration and purity of RNA were detected by enzyme-labeled instruments, brain tissues were stored separately at −80°C in refrigerators.

Complementary DNA (cDNA) was reversely transcribed according to primescript™ RT reagent kit with gDNA eraser (perfect real time) instruction. The relative expression levels of target genes, including Ghrelin, Aggf1, and reduced glyceraldehyde-phosphate dehydrogenase (GAPDH) were detected according to TB Green® premix ex taq™ (tli RNaseH plus) instructions. Table 3 shows target gene primers. GAPDH gene was adopted as the internal reference to detect the relative expression levels of target genes according to the equation 2^−ΔΔCt.^

**Table 3.** Information about quantitative detection primers of target genes

Gene name	Direction	Sequence (5’→3’)	Product size (bp)
Ghrelin	F	AGCCCAGCAGAGAAAGGAAT	206
	R	GAAGAAACTTTCCCAGGGCC	
Aggf1	F	CAAGCTCCTTGGGCGATTTCA	161
	R	GCATAACTCCCGCTGTCTATGT	
GAPDH	F	AATGGATTTGGACGCATTGGT	213
	R	TTTGCACTGGTACGTGTTGAT	

### 2.7. Western blot detection

100 mg of rat brain tissues was extracted and added with 1 mL of radio immunoprecipitation assay solution for homogenization. After that, it was centrifuged at 4°C and 12000 rpm for 20 minutes. Then, supernatant was taken and the protein concentration of samples was detected by bicinchoninic acid (BCA) method. Besides, the protein concentration of samples was calculated by drawing standard curves. After 5× sodium dodecyl sulfate polyacrylamide gel electrophoresis (SDS-PAGE) buffer solution was added, it was heated at 100°C for 5 minutes. Samples were loaded after SDS-PAGE buffer solution was prepared. After electrophoresis, the glue was cut and the membrane was transferred to polyvinylidene fluoride. Next, it was sealed by the added confining liquid containing 5% of skimmed milk powder for 1 hour. Later on, it was mixed with the solution containing diluted first anti-bodies, including Ghrelin, Aggf1, Akt, p-Akt, caspase-3, and β-actin. After that, it was placed and incubated overnight at 4°C in refrigerator. After the membrane was washed with tris buffered saline tween (TBST), it was incubated by the second antibody marked with horseradish peroxidase (HRP) at room temperature for 2 hours. After the membrane was washed with TBST, ECL color liquid was added for image developing. In addition, national institutes of health (NIH) image J software was adopted in the gray scale of target protein bands. β-actin was utilized as the internal reference to calculate the relative expression levels of target genes.

### 2.8. Oxidative stress index detection

According to GSH (60342ES10,10 g) and GSSG (60341ES03,1 g) kit instruction, double antibody sandwich ABC-ELISA method was used to detect GSH and GSSG contents in rat cerebral tissues. Anti-rat GSH and GSSG monoclonal antibodies were coated on enzyme labeled plate. GSH in standards and samples are combined with monoclonal antibody. Besides, Biotinylated anti-rat GSH was added to form immune complexes attached to the plate. Streptavidin marked with HRP was combined with biotin. After that, substrate working solution was added to show blue color, and sulfuric acid was finally added as the termination solution. Optical density (OD) was measured at 450 nm, and GSH concentration was proportional to OD. GSH concentration in the specimen could be calculated by drawing standard curves. The value fetched at the 30^th^ second was set to be A1. Besides, the value fetched after samples were placed at room temperature silently for 5 minutes was set to be A2. A1 and A2 were adopted in the two equations of sample ΔA(A2-A1)/standard ΔA(A2-A1) × standard concentration × dilution ratio, respectively, to detect the contents of GSH and GSSG. The process of preparing standard was as follows. 0.5 mL of distilled water was added and mixed evenly into 4000 pmoL/mL before use. The number of standard tubes was set to be 8 and each tube was added with 200 uL specimen diluent. 200 uL standard solution with a concentration of 4000 pmoL/mL was added into the 1^st^ tube and mixed evenly. After that, 200 uL standard solution was sucked out and transferred to the 2^nd^ tube by sample injector. Multiple dilution was repeated in the same way. 200 uL standard solution was removed from the 7^th^ tube and the 8^th^ was utilized as the blank control [14].

### 2.9. Statistical processing

Excel was adopted in data statistics and diagram drawing. All statistical processes were achieved by statistical product and service solutions software (SPSS) 19.0. One-way variance analysis was utilized in analyzing the differences between groups. Besides, *P* < 0.05 indicated that the differences showed statistical meaning.

## 3. Results

mRNA expression levels of Ghrelin and Aggf1 in cerebral tissues of the rats in different groups were investigated, including sham group, SAH group, negative control group, Ghrelin silence group, and Aggf1 silence group. Besides, rat neurologic dysfunction scores and the influences of rat brain water content were also explored. Western blotting was adopted to detect the expression levels of Ghrelin, Aggf1, p-Akt, and caspase-3 in rat cerebral tissues. GSH contents, GSSG contents, and the differences in ratio of GSH/GSSG in rat cerebral tissues were compared to analyze the influences of silence Ghrelin and Aggf1 on rat cerebral oxidative stress, which confirmed that Ghrelin and Aggf1 could resist oxidation and inhibit early cerebral injury after SAH. The results of the research provided the reference for the prevention and treatment of early cerebral injury after clinical pons SAH.

### 3.1. Analysis of rat survival

All rats in sham group survived and the mortality was 0% (0/20). In model group, a rat died of cardiac arrest during modeling process, and another rat died 24 hours after modeling. The mortality of rats was 10% (2/20). In negative control group, 3 rats died of respiratory or cardiac arrest during blood injection process. The mortality of rats amounted to 15% (3/20). In Ghrelin^(-/-)^ group, 3 rats died of respiratory or cardiac arrest during blood injection process, and 2 died 24 hours after modeling. The mortality reached 25% (5/20). In Aggf1^(-/-)^ group, 4 rats died of respiratory or cardiac arrest during blood injection process. The mortality reached 20% (4/20). When some animals were dead, the same number of rats were added into the groups in which some rats died.

### 3.2. Detection in mRNA expression levels of rat brain tissues Ghrelin and Aggf1 by RT-qPCR

**Figure 1.**
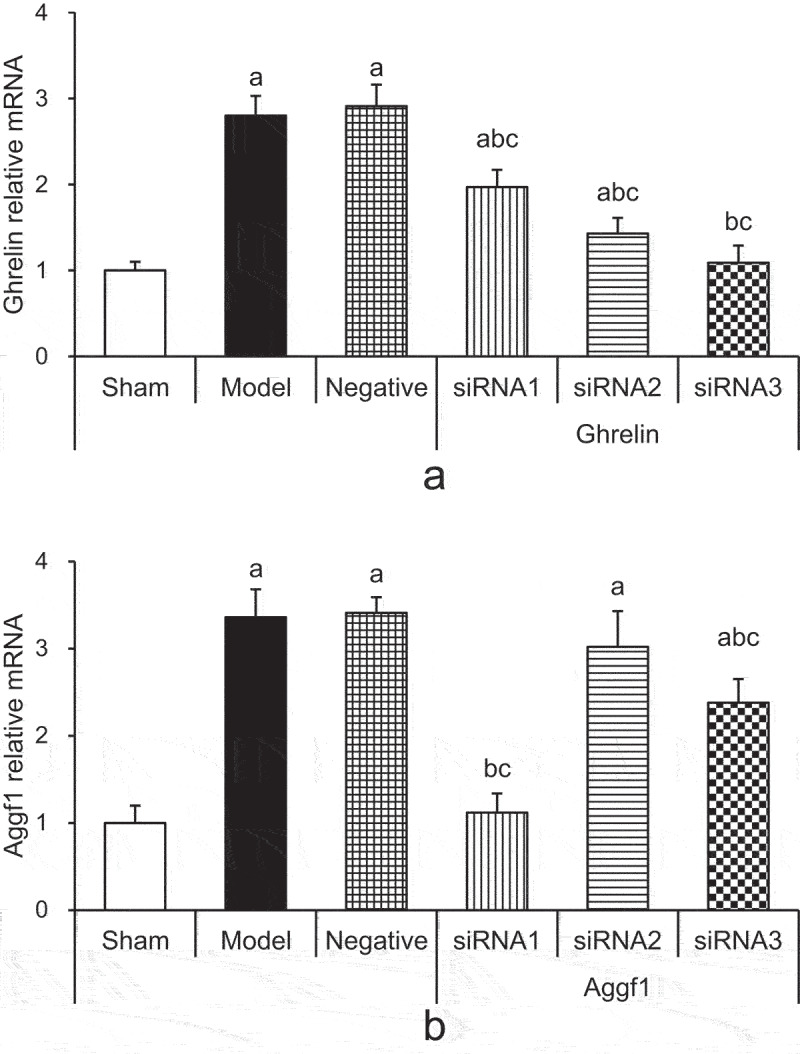
Comparison of mRNA expression levels of target genes in rat brain tissues of each group after silence Ghrelin and Aggf1. (a showed Ghrelin mRNA expression levels, and b demonstrated Aggf1 mRNA expression levels. The comparison with sham group indicated ^a^*P* < 0.05, the comparison with model group revealed ^b^*P* < 0.05, and the comparison with negative group showed ^c^*P* < 0.05).

According to Figure 1(a), the mRNA relative expression levels of rats in model group, negative group, Ghrelin siRNA1 group, and Ghrelin siRNA2 group were enhanced significantly compared with those of rats in sham group (*P* < 0.05). In contrast, the mRNA expression levels of rats between sham group and Ghrelin siRNA3 group showed no obvious differences (*P* > 0.05). Besides, the Ghrelin mRNA relative expression levels of rats in Ghrelin siRNA1, Ghrelin siRNA2, and Ghrelin siRNA3 groups were significantly reduced compared with those of rats in model group and negative group (*P* < 0.05). Therefore, Ghrelin siRNA3 fragments were selected for subsequent experiments.

As Figure 1(b) demonstrated, Aggf1 mRNA relative expression levels of rats in model group, negative group, Aggf1 siRNA2 group, and Aggf1 siRNA3 group were obviously increased compared with those of rats in sham group (*P* < 0.05). In contrast, the Aggf1 mRNA relative expression levels of rats in sham group and Aggf1 siRNA1 group demonstrated no significant differences (*P* > 0.05). Compared with those in model group and negative group, Aggf1 mRNA relative expression levels of rats in Aggf1 siRNA1 and Aggf1 siRNA3 groups were obviously decreased (*P* < 0.05), while showed no significant differences compared with those of rats in Aggf1 siRNA2 group (*P* > 0.05). Therefore, Aggf1 siRNA1 fragments were selected for subsequent experiment.

### 3.3. Influences of silence Ghrelin and Aggf1 on rat neurologic dysfunction

**Figure 2.**
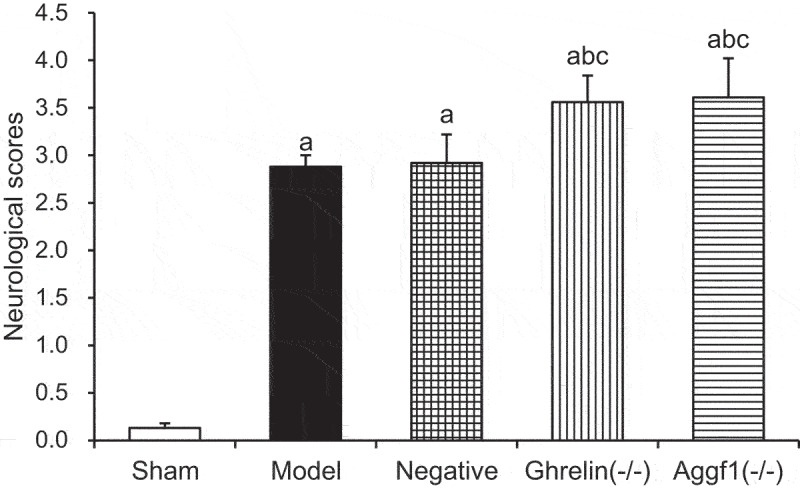
Comparison of scores of rat neurologic dysfunction in each group. (The comparison with sham group indicated ^a^*P* < 0.05, the comparison with model group revealed that ^b^*P* < 0.05, and the comparison with negative group showed that ^c^*P* < 0.05).

According to Figure 2, the scores of rat neurologic dysfunction in model group, negative group, Ghrelin^(-/-)^ group, and Aggf1^(-/-)^ group were significantly increased compared with that in sham group (*P* < 0.05). Compared with those in model group and negative group, the scores of rat neurologic dysfunction in Ghrelin^(-/-)^ group and Aggf1^(-/-)^ group were also obviously enhanced (*P* < 0.05). Besides, there was no significant differences between the scores of rat neurologic dysfunction in Ghrelin^(-/-)^ group and Aggf1^(-/-)^ group (*P* > 0.05).

### 3.4. Influences of silence Ghrelin and Aggf1 on rat cerebral water content

**Figure 3.**
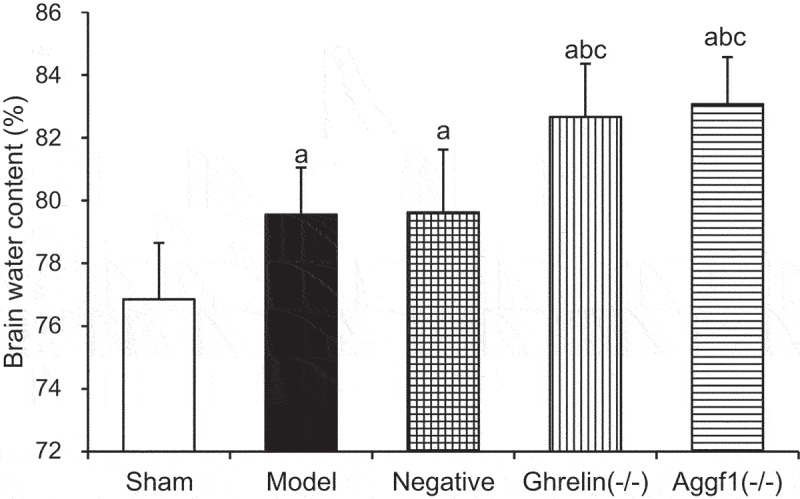
Comparison of rat cerebral water contents in each group. (The comparison with sham group indicated ^a^*P* < 0.05, the comparison with model group showed ^b^*P* < 0.05, and the comparison with negative group revealed that ^c^*P* < 0.05).

According to Figure 3, the rat cerebral water contents in model group, negative group, Ghrelin^(-/-)^ group, and Aggf1^(-/-)^ group were obviously increased compared with that in sham group (*P* < 0.05). Compared with those in model group and negative group, the rat cerebral water contents in Ghrelin^(-/-)^ group and Aggf1^(-/-)^ group were also significantly enhanced (*P* < 0.05). In contrast, the comparison of the rat cerebral water contents in Ghrelin^(-/-)^ group and Aggf1^(-/-)^ group demonstrated no obvious differences (*P* > 0.05).

### 3.5. Comparison of expression levels of Ghrelin, Aggf1, p-Akt, and caspase-3 in cerebral tissues of rats in each group

**Figure 4.**
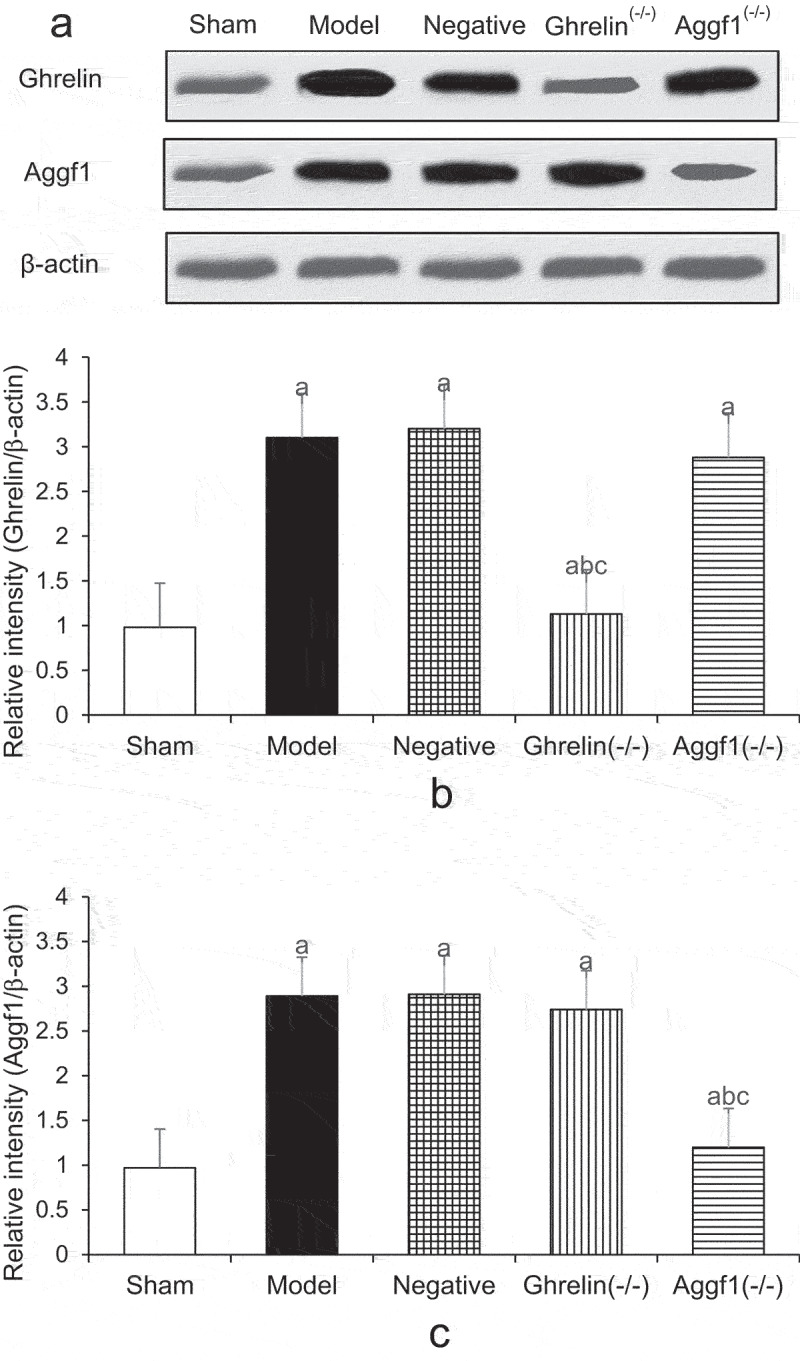
Comparison of Ghrelin and Aggf1 protein expression levels of rat brains in five groups (a showed Western blotting, b displayed Ghrelin protein expression levels, and c indicated Aggf1 protein expression levels. The comparison with sham group demonstrated *P* < 0.05, the comparison with model group revealed ^b^*P* < 0.05, and the comparison with negative group suggested ^c^*P* < 0.05).

**Figure 5.**
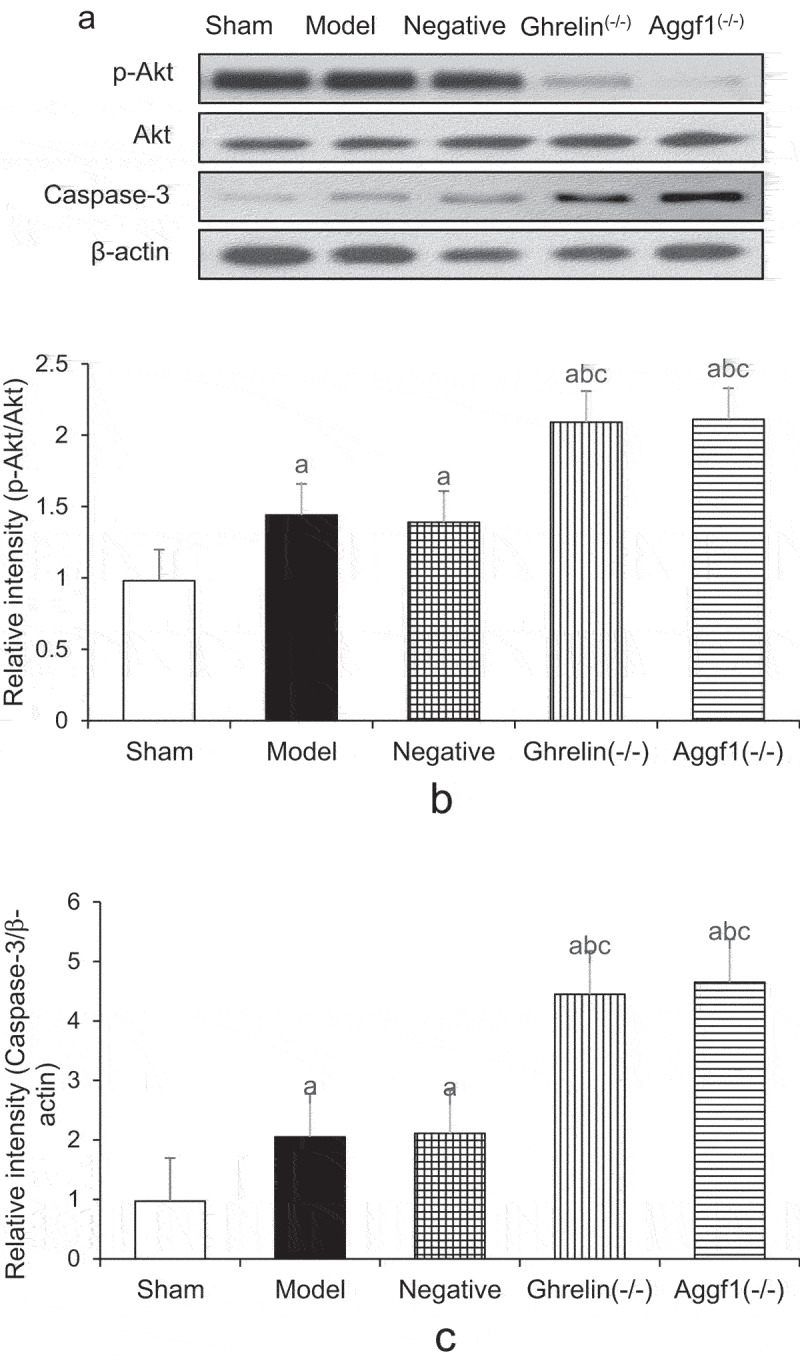
Comparison of p-Akt and caspase-3 expression levels of rat brains in five groups (a showed Western blotting, b displayed p-Akt protein expression levels, and c indicated caspase-3 protein expression levels. The comparison with sham group demonstrated *P* < 0.05, the comparison with model group revealed ^b^*P* < 0.05, and the comparison with negative group suggested ^c^*P* < 0.05).

Figures 4 and 5 demonstrate the comparison of the expression levels of Ghrelin, Aggf1, p-Akt, and caspase-3 in rat cerebral tissues detected by Western blotting. According to Figures, Ghrelin protein expression levels in model, negative, and Aggf1 (-/-) groups were significantly increased compared with that in sham group (*P* < 0.05). In contrast, Ghrelin protein expression level in Ghrelin (-/-) group was obviously lower than those in model group, negative group, and Aggf1 (-/-) group (*P* < 0.05). In addition, the comparison of Ghrelin protein expression levels in model group, negative group, and Aggf1 (-/-) group showed no significant differences (*P* < 0.05). Aggf1 protein expression levels in model group, negative group, and Ghrelin (-/-) group were obviously higher than that in sham group (*P* < 0.05). In contrast, Aggf1 protein expression level in Aggf1 (-/-) group was remarkably lower than those in model group, negative group, and Ghrelin (-/-) group (*P* < 0.05). In addition, Aggf1 protein expression levels in model group, negative group, and Ghrelin (-/-) group showed no significant differences (*P* < 0.05).

### 3.6. Influences of silence Ghrelin and Aggf1 on rat brain oxidative stress

**Figure 6.**
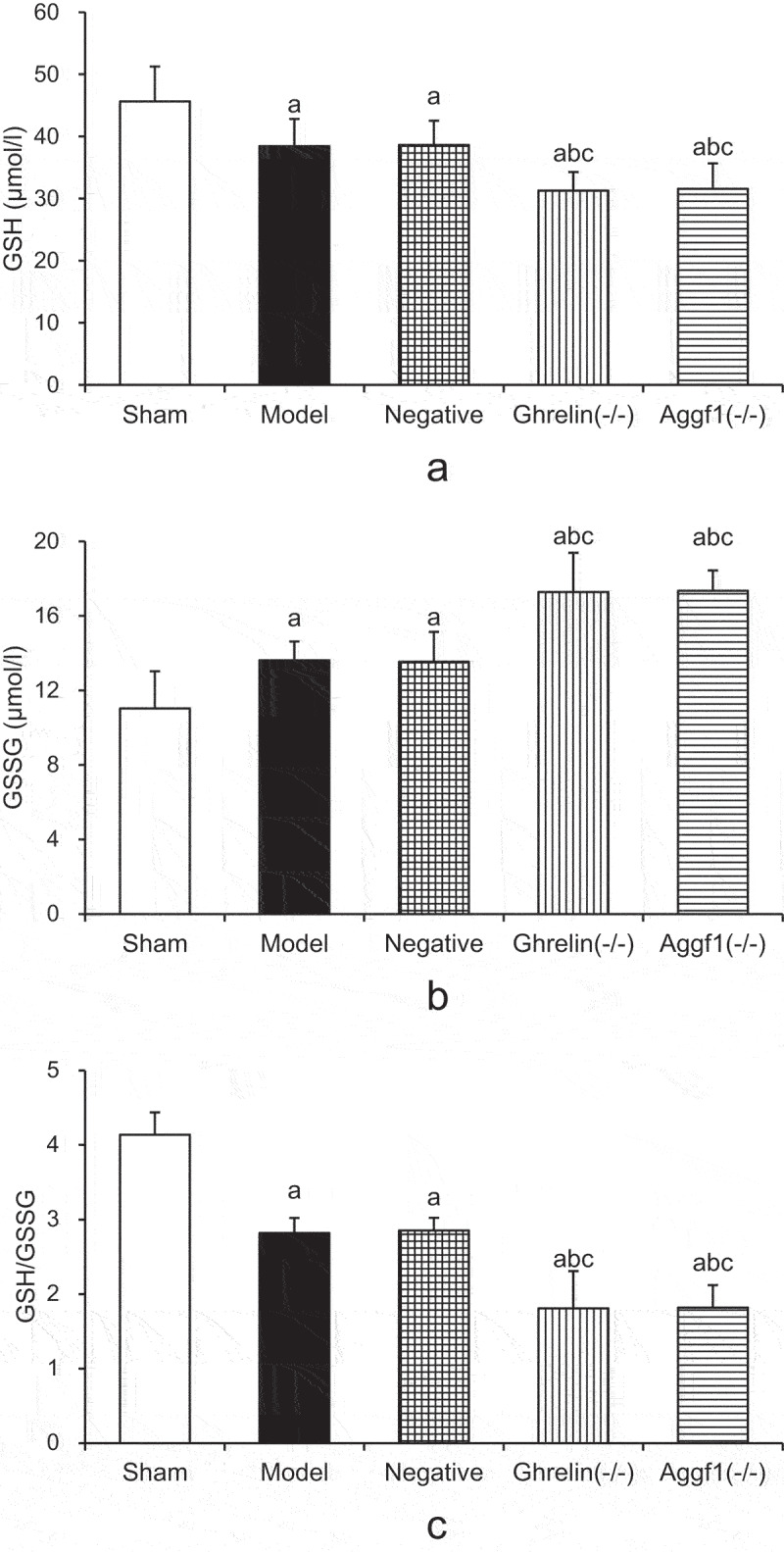
Comparison of GSH, GSSG, and GSH/GSSG levels of rat brains in each group. (a showed the comparison of GSH contents, b demonstrated the comparison of GSSG contents, and c presented GSH/GSGG ratio values. The comparison with sham group indicated ^a^*P* < 0.05, the comparison with model group revealed ^b^*P* < 0.05, and the comparison with negative group demonstrated ^c^*P* < 0.05.).

According to Figure 6, GSH contents and GSH/GSSG ratio values of rat brain tissues in model group, negative group, Ghrelin^(-/-)^ group, and Aggf1^(-/-)^ group were significantly decreased compared with those in sham group, while GSSG contents in these groups were enhanced obviously (*P* < 0.05). Compared with those in model group and negative group, GSH contents and GSH/GSSG ratio values of rat brain tissues in Ghrelin^(-/-)^ group and Aggf1^(-/-)^ group were also obviously reduced, while GSSG contents in the two groups were increased (*P* < 0.05). Besides, GSH contents, GSSG contents, and GSH/GSSG ratio values of rat brain tissues between model group and negative group, and between Ghrelin^(-/-)^ group and Aggf1^(-/-)^ group demonstrated no significant differences (*P* > 0.05).

## 4. Discussion

SAH is a cerebrovascular disease causing mortality and disability and threatening patients’ lives. Cerebral vascular spasm and early cerebral injury are the main clinical manifestations of SAH patients. Some researchers investigated the incidence mechanism of early cell injury after SAH and its relevant capillary vessel spasm and neurovascular injury, and discovered that scutellarin showed good effects on treating SAH. Besides, intracerebral thrombus of experimental rats was obviously reduced to mitigate cerebrovascular injury [6]. Early cerebral injury is the major factor causing the poor prognosis of SAH patients [15]. The pathophysiological processes of early cerebral injury after SAH are very complex and related to autophagy, apoptosis, inflammation reaction, and oxidative stress [3]. Aggf1 is widely expressed in brains, eyes, hearts, and other human tissues, organs, and cells. Researchers found out that Aggf1 showed significant effects on promoting angiogenesis. A large number of studies confirmed that Aggf1 responded to inflammations and maintained vascular integrity [16,17]. What’s more, Aggf1 blocked endothelial activation by phosphatidylinositol 3-kinase (PI3K)/Akt/nuclear factor kappa-B signal pathway and inhibited inflammation reaction [18]. Zhu et al. (2018) [18] carried out intravascular perforation and modeling of SAH on male SD rats to investigate the influences of G-patch and FHA structure domain 1 on the neurologic functions of rats after SAH, which verified that Aggf1 angiogenic factors could inhibit inflammatory actions and maintain vascular integrity of non-nervous system diseases. In addition, Aggf1 angiogenic factors could alleviate neuroinflammation and blood–brain barrier damage. Based on the above results, the influences of Aggf1 on rat cerebral oxidative stress were investigated, and the action mechanism of Aggf1 in avoiding early cerebral injury after SAH was further analyzed. Ghrelin is a polypeptide with a wide range of biological functions. In addition, some relevant studies verified that Ghrelin could resist inflammations to some degree. Hao et al. (2014) [19] explored whether Ghrelin could reduce early cerebral injury after SAH and analyzed the influences of Ghrelin on rat mortality, nervous system scores, cerebral edema, apoptosis, and the expression levels of p-Akt and dissected caspase-3 protein after SAH. They found out that Ghrelin could significantly reduce neuronal cell apoptosis and cerebral edema of rats after SAH. Relevant studies revealed that Ghrelin could inhibit proinflammatory mediators and reduce SAH-induced oxidative cerebral injury by avoiding the consumption of endogenous antioxidants caused by SAH [20]. Based on the results, the influences of Ghrelin on rat cerebral oxidative stress and brain water content were analyzed, and the action mechanism of Ghrelin in avoiding early cerebral injury after SAH was further investigated. Optic chiasm pretumor reservoir injection method was adopted to prepare SAH rat model. In addition, Ghrelin and Aggf1 were designed and injected, and the action mechanism of silence Ghrelin and Aggf1 genes in rat early cerebral injury and oxidative stress reaction was analyzed. Relevant studies indicated that Prx-1 siRNA silencing would up-regulate intracellular reactive oxidative species (ROS) level and the expression of p-Akt protein [21].

Excessive production of oxygen free radicals is the main pathophysiological cause of early cerebral injury, which results in obvious neurologic dysfunction and aggravated cerebral edema. Severe cerebral injury usually occurs at the early stage of SAH, which may be one of the causes of high morality among patients with early SAH. In addition, it is more obvious that neurologic dysfunction and cerebral dysfunction of rats are aggravated after SAH. PI3K/Akt signal pathway is an essential way to inhibit apoptosis and gets involved in the process of protecting central nervous system. According to relevant studies, PI3K/Akt signal pathways played a significant role in nerve protection after SAH by regulating neuronal cell apoptosis [22]. Some researchers observed the influences of Growth hormone releasing peptide on the integrity of blood–brain barriers, brain water content, neuronal cell apoptosis, and p-Akt and anti-apoptosis proteins in PI3K/Akt signal pathways of mice with injured brains. The results indicated that Ghrelin could alleviate cerebral injury of mice, which might be caused by the activation of PI3K/Akt signal pathways [23]. What’s more, Ghrelin could mitigate cerebral edema, avoid neuronal cell apoptosis, improve the activity of superoxide dismutase (SOD), and reduce malondialdehyde content. In addition, it could enhance the expressions of p-Akt and Bcl-2 while reduce the expression of Bax. The reduction in GSH content and the increased in GSSG content are the important markers of oxidative stress injury. The decline of GSH content and the increase of GSSG content in cerebral tissues of SAH rats were more obvious [24].

The results of the research demonstrated that the expression levels of p-Akt and caspase-3 proteins were significantly increased 24 hours after SAH modeling. After Ghrelin or Aggf1 was silenced, the increase in p-Akt and caspase-3 in cerebral tissues of SAH rats became abnormal after SAH. The differences showed that PI3K/Akt signal pathway was activated 24 hours after SAH, and the silence of Ghrelin and Aggf1 promoted the activation of PI3K/Akt signal pathway and the apoptosis of neuronal cells to avoid early cerebral injury after SAH to some extent.

## 5. Conclusion

The protection mechanism of early cerebral injury after SAH was investigated, which revealed that cerebral nerve injury of model rats became obvious after the silence of Ghrelin and Aggf1. Besides, brain water content was increased, oxidative stress level was enhanced, and cerebral injury was aggravated. The results demonstrated that Ghrelin and Aggf1 protected brains in early cerebral injury after SAH. The disadvantages of the research included small sample size and no in-depth investigation into the signal pathway of Ghrelin and Aggf1, which needed to be further researched and discussed in future studies.

## Highlights

After the silence of Ghrelin and Aggf1, rat neurological impairment level increased.

After silence of Ghrelin and Aggf1, water content in cerebral tissues increased.

Inhibition of Ghrelin and Aggf1 expression avoids some early cerebral injuries.
